# Musculoskeletal manifestations of diabetes mellitus – an update^✰^

**DOI:** 10.1016/j.clinme.2025.100498

**Published:** 2025-08-10

**Authors:** Harry Ward, Ali S. Jawad

**Affiliations:** a Academic clinical fellow in Rheumatology (ST2), Rheumatology department, Royal London Hospital, London E1 4DG, UK; b Consultant Rheumatologist, Rheumatology department, Royal London Hospital, London E1 4DG, UK

**Keywords:** Diabetes, Musculoskeletal, Neuroarthropathy, Fibrosing syndrome, Sarcopenia, Muscle infarction, Amyotrophy, Osteoporosis, Osteoarthritis, Stiff person syndrome, Diffuse idiopathic skeletal hyperostosis, Rheumatoid arthritis

## Abstract

Diabetes mellitus is an increasingly prevalent condition, with an estimated 1.3 billion people projected to be affected by 2050, and can reduce life expectancy by up to 20 years. Musculoskeletal complications are present in more than half of patients with diabetes and lead to significant morbidity and disability. Diabetes increases chronic inflammation, reduces anabolism and compromises blood supply to bones, muscles and joints. This can cause fibrosis of tendons and joints, impaired quality of bone and muscle production and, in some instances, local joint destruction. The risk is further increased in patients with overlapping metabolic syndrome. Musculoskeletal complications promote a sedentary lifestyle and worsen the trajectory of diabetes management. Here, we provide an update on the musculoskeletal manifestations of diabetes and their treatments since our review in 2015.

## Highlights

• Musculoskeletal conditions associated with diabetes are under-appreciated and contribute significant morbidity for patients. Generally, improved glycaemic control, a balanced diet with reduced saturated fat intake and regular exercise reduce the risk of these conditions.
• Fibrosing musculoskeletal conditions and internal organ fibrosis are more common in diabetes.
• Patients with type 2 diabetes are at higher risk of fracture despite a normal bone mineral density. This risk is better predicted by FRAXplus and QFracture risk prediction tools.
• Patients with type 2 diabetes presenting with metabolic syndrome have an increased risk of musculoskeletal complications due to chronic inflammation, oxidative stress and obesity sarcopenia.
• Type 1 diabetes is associated with other autoimmune conditions such as stiff person syndrome and rheumatoid arthritis.

## Introduction

Diabetes mellitus affects 529 million people worldwide. With a worsening diet and rising BMI in the population, it is projected to affect 1.31 billion people by 2050.^1^ Musculoskeletal complications are equally common, occurring in 58% of patients, but less appreciated (Table 1).^2^ Diabetes can affect joints, tendons, muscles and bones. This can lead to significant morbidity and disability, which subsequently worsens general health, and particularly cardiovascular outcomes, due to reduced mobility and weight gain. Here, we provide an update on the musculoskeletal manifestations of diabetes and their treatments since our review in 2015.^3^

**Table 1:** Outline of musculoskeletal manifestations of diabetes mellitus.

Joint disorder	Neuroarthropathy	
Soft tissues conditions	Fibrosing syndromes	Limited joint mobility Dupuytren’s contracture Stenosing tenosynovitis Carpal tunnel syndrome Frozen shoulder Peyronie’s disease Sclerederma
Muscle conditions	Sarcopenia Muscle infarction Amyotrophy	
Bone disorder	Osteoporosis	
Disease associations	Osteoporosis Stiff person syndrome DISH Rheumatoid arthritis	

## Musculoskeletal manifestation of diabetes

### Charcot neuroarthropathy

**Fig. 1:**
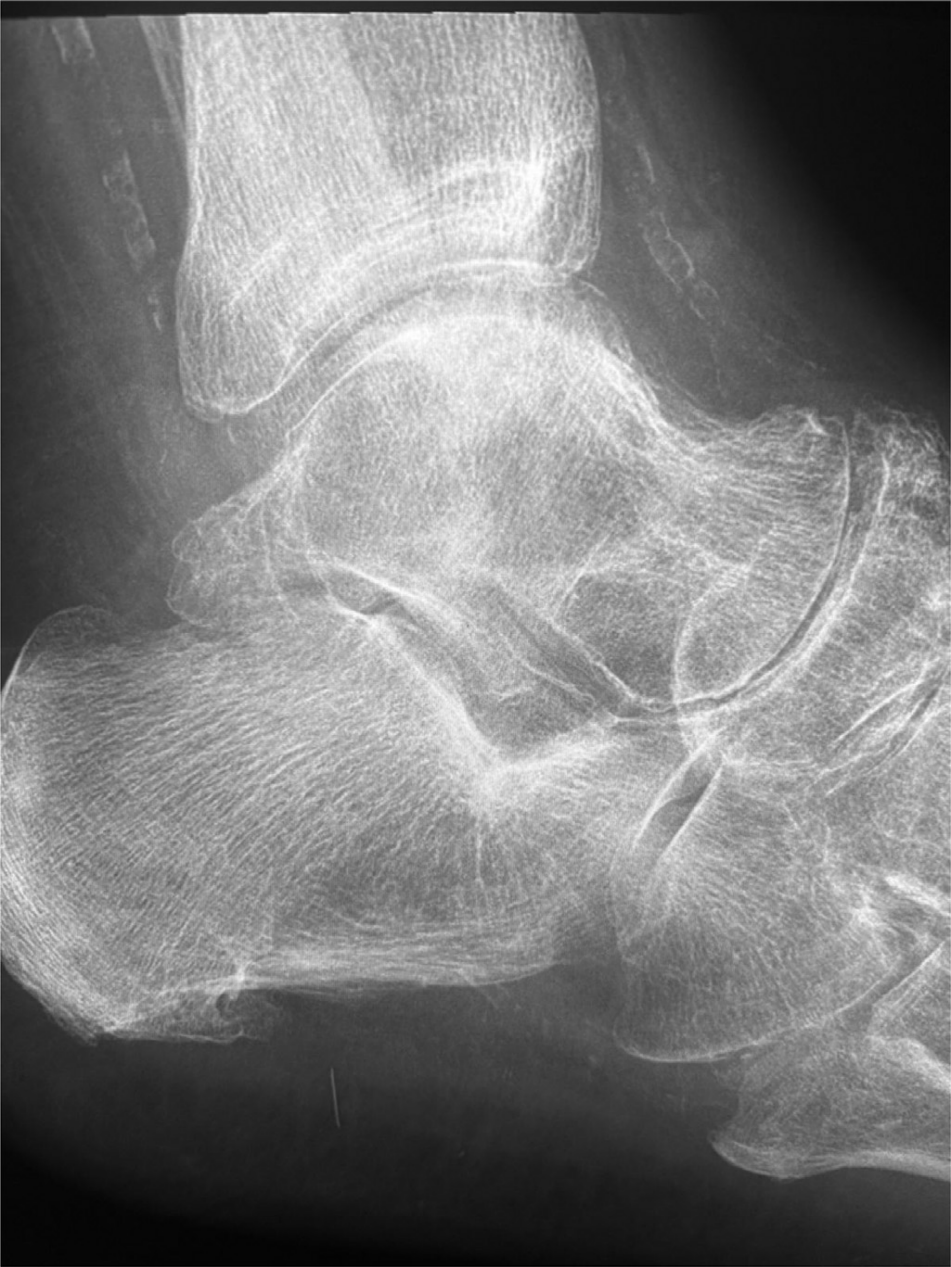
X-ray of the ankle of a patient with long-standing type 1 diabetes and peripheral neuropathy, showing bone resorption and fragmentation especially in the dome of the talus (osteonecrosis). Note the calcification of the tibialis posterior and the dorsalis pedis arteries.

Charcot neuroarthropathy, first described by Jean-Martin Charcot in 1868 in syphilis patients with tabes dorsalis, is the progressive deformity and restructuring of the ankle and foot secondary to neuropathy in advanced diabetes. In diabetes, the joints affected in descending frequency are the ankles, metatarsophalangeal and mid-tarsal joints. One theory is that damage to peripheral nerves increases blood flow and bone resorption, fragmentation and disorganisation. Combined with muscle atrophy due to denervation, poor sensation and microfractures, the ankle and foot slowly restructure (Fig. 1). Early diagnosis is hindered by a lack of symptoms. Initially, the foot may be slightly red, warm and swollen and MRI may show microfractures and bone marrow oedema. Foot ulceration occurs in up to a third of patients with diabetes and neuropathy. Lower limb complications of diabetes are among the WHO top ten conditions in terms of years lived with disability. Ischaemia, neuropathy and ulceration predispose to septic arthritis. Annual foot checks facilitate early referral to orthotics and prevent ulceration and infection. Above all, tight glycaemic control is paramount to prevent the development and progression of neuropathy.

### Fibrosing syndromes

Diabetes can predispose to a host of fibrosing syndromes. The underlying pathophysiology is thought to be multifaceted. Chronic hyperglycaemia results in the accumulation of advanced glycation end-products (AGEs), which drive chronic inflammation, oxidative stress and fibroblast activation. Chronic hyperglycaemia also results in microangiopathy. The resultant poor blood supply causes chronic hypoxia and inflammation.

**Fig. 2:**
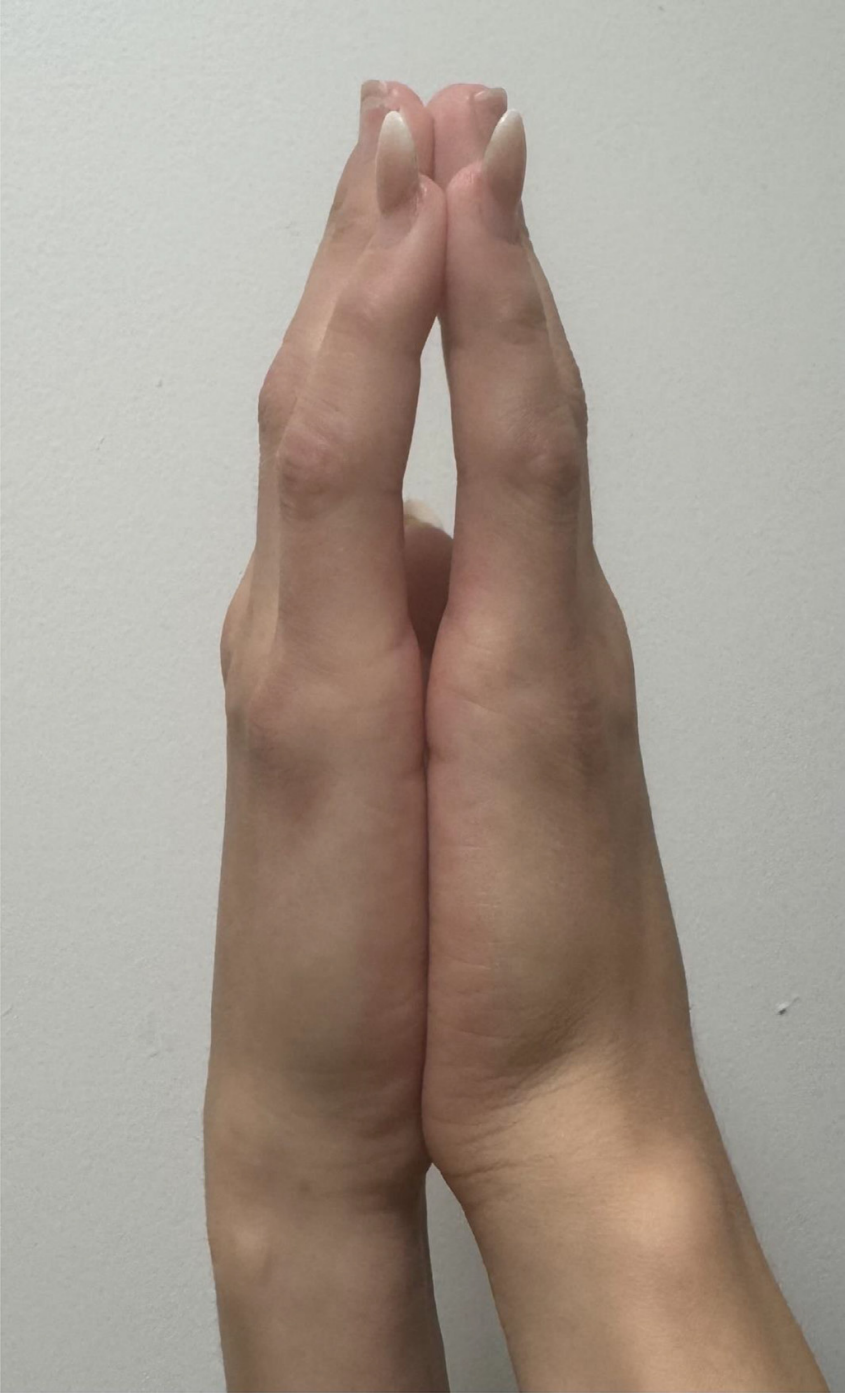
A 35-year-old female with T1D for 31 years showing the prayer’s sign due to limited joint mobility. She is also a smoker.

Diabetes cheiroarthropathy is characterised by thickening of skin and stiffness in the fingers. Clinically, patients have a positive ‘prayer sign’. This is usually seen in people with long-standing diabetes (Fig. 2).

Dupuytren’s contracture is the result of palmar fascia thickening in the palm, usually affecting the third or fourth finger and can be bilateral. It is more common in type 2 diabetes mellitus (T2DM) than in T1DM and in those with worse glycaemic control.^4^

Trigger finger occurs when the tendon sheath becomes thickened (stenosing tenosynoviopathy), resulting in catching or locking of the finger.

Carpal tunnel syndrome develops due to the narrowing of the carpal tunnel and subsequent compression of the median nerve. This results in loss of sensation in the lateral half of the hand and atrophy of hand muscles. People with diabetes are twice as likely to present with advanced disease compared to the general population.^5^

Peyronie’s disease, which is the gradual deformity of the penis due to fibrous plaque formation in the tunica albuginea, is four to five times more common in people with diabetes.^6^

Frozen shoulder, or adhesive capsulitis, is caused by fibrosis of the shoulder capsule. Diabetic patients tend to have a long ‘frozen’ duration and worse recovery during the ‘thawing’ stage.^7^

Sclerederma diabeticorum presents with thickening of the skin on the back and neck in advanced T2DM. This is due to mucin deposits in thickened collagen bundles in the skin. Case reports show inconsistent benefit with a wide range of treatments, from UV therapy to corticosteroids to immunosuppressants.

Generally, treatment for musculoskeletal fibrosing conditions is physiotherapy, rest and splinting. Injection of collagenase or corticosteroids can be beneficial. Severe cases may require surgical removal or incision of the fibrous tissue to restore function. Interestingly, DPP-4 inhibitors and GLP-1 agonists have been correlated with reduced organ fibrosis; however, clinical trials have yet to be conducted.^8^

## Muscle involvement

### Sarcopenia

Advanced diabetes results in sarcopenia. There are multiple causes of this. Chronic inflammation reduces muscle fibre regeneration. Impaired insulin signalling reduces its effect as an anabolic hormone on muscle. Mitochondrial dysfunction, oxidative stress and microangiopathy also worsen the function of existing muscle fibres.^9^ Sarcopenia results in poor postural stability, falls and frailty.

### Diabetic muscle infarction

Diabetic muscle infarction typically occurs in patients with advanced diabetes. It presents with sudden swelling and muscle pain without weakness. These patients tend to have raised inflammatory markers and a normal creatine kinase. MRI shows muscle oedema and necrosis. It is thought that this is due to progressive microangiopathy and atherosclerosis. Treatment is conservative.

### Diabetic amyotrophy

Diabetic amyotrophy presents in older patients with asymmetric gradual-onset severe thigh pain and weakness. Generally, these patients have normal inflammatory markers and creatine kinase. Electromyography (EMG) and nerve conduction studies (NCS) show muscle denervation and axonal degeneration. The underlying pathophysiology is felt to be a microvasculopathy and nerve infarction. Interestingly, converse to other complications of diabetes, risk factors for this condition are rapid correction of sugars and tight glycaemic control. A Cochrane review in 2017 shows that intravenous methylprednisolone reduced pain but did not speed up recovery, and there was no evidence to support the use of intravenous immunoglobulin.^10^

## Bone disorders

### Osteoporosis

T1DM is a known cause of reduced bone mineral density. T2DM, however, increases fracture risk without decreasing bone mineral density. There are several factors at play. Accumulation of AGEs in bone promotes inflammation and oxidative stress, particularly in patients with overlapping metabolic syndrome. These, along with insulin resistance, reduce bone turnover. Furthermore, hyperglycosuria results in urinary calcium loss, and diabetic nephropathy can result in renal mineral bone disease. Some anti-diabetic medications increase fracture risk, such as thiazolidinediones and insulin. Conflicting data on SGLT2 inhibitors initially suggested an increased fracture risk, but this has not been corroborated more recently.^11^ Crucially, people with diabetes are also at a greater risk of falling due to visual impairment from retinopathy, neuropathy, sarcopenia, cardiovascular disease and postural hypotension. An addition to the traditional FRAX calculation tool, FRAXPlus, has improved fracture risk prediction in patients with T2DM. Alternatively, QFracture incorporates the presence of diabetes and the risk of falls as separate predictors. More recent biomarker studies have shown that HbA1c and IGF-1, and not bone turnover markers, correlate with fracture risk.^12^

There are no head-to-head trials for osteoporosis treatment in diabetes. However, interestingly, denosumab reduced the incidence of diabetes in osteoporotic patients compared with zoledronate, suggesting an additional role in glucose metabolism.^13^

## Diseases associated with diabetes

### Osteoarthritis

Nearly half of T2DM patients have arthritis. Even when factoring in obesity, this association remains.^14^ There is even an association with progression of erosive hand osteoarthritis.^15^ GLP-1 agonists show promising pre-clinical data with reduced macrophage activation in mouse models.^8^

### Stiff person syndrome

Stiff person syndrome is a neurological autoimmune condition targeting GABAergic neurons, which results in muscle hyperexcitability. It presents with stiff muscles and painful spasms. The most common autoantibody is anti-GAD65. This is also seen in T1DM where antibodies target GABAergic neurons innervating the pancreas. In fact, 60% of patients with stiff person syndrome develop T1DM, and people with T1DM are up to 100 times more likely to develop stiff person syndrome.^16^ It is very unusual for patients without anti-GAD antibodies to develop diabetes. Other subtypes of stiff person syndrome include genetic and paraneoplastic syndromes.

### DISH

**Fig. 3:**
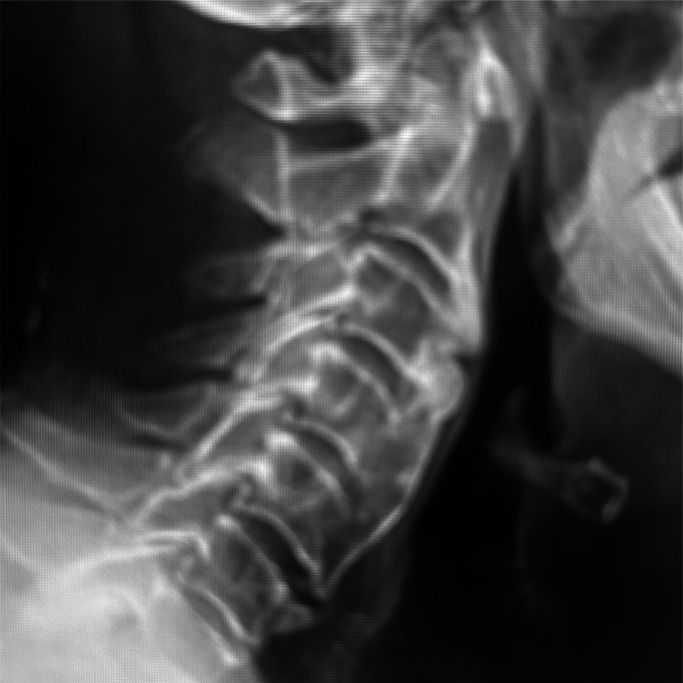
X-ray of the cervical spine of a patient with type 2 diabetes showing flowing anterior osteophytes over four contiguous vertebrae with preservation of intervertebral disc space without significant degenerative intervertebral disease. There is absence of facet and costovertebral joint ankyloses.

Diffuse idiopathic skeletal hyperostosis (DISH) presents with calcification and ossification of ligaments and tendons, typically in patients over 50 years old. The diagnosis requires bridging osteophytes in four adjacent thoracic vertebrae (Fig. 3). It is associated with T2DM and metabolic syndrome. It most commonly affects the spine, presenting with stiffness and pain, but rarely presents with symptoms from compression of surrounding structures. Treatment initially involves physiotherapy and pain relief. Surgery may be required if there is compression of surrounding structures. Unfortunately, the diagnostic criteria mean that this condition is often diagnosed late, making treatment difficult. Bisphosphonates are not effective in our experience.

### Rheumatoid arthritis

T1DM patients are almost five times more likely to develop anti-CCP-positive rheumatoid arthritis. These patients share the polymorphism 620W in the PTPN22 allele.^17^ GWAS studies comparing the two conditions have found correlated genetic loci and suggest a causal relationship for the shared genetics.^18^

T2DM patients are up to 50% more likely to have rheumatoid arthritis.^19^ However, this association is probably related to steroid use, obesity and chronic inflammation in rheumatoid arthritis rather than a predisposing genetic background. In fact, a recent study showed that T2DM patients had a protective genotype for rheumatoid arthritis.^19^

## Conclusion

Multidisciplinary management of diabetes has improved detection of complications and overall outcomes in diabetes. Annual foot checks and retinal screening are good examples of this. However, musculoskeletal complications remain relatively overlooked. More vigilance, particularly in overlapping metabolic syndrome, may allow earlier escalation to more appropriate therapies, such as a GLP-1 agonist, but also allow patients to implement proactive lifestyle changes. This may help to prevent the spiral of disability from musculoskeletal complications which results in weight gain, worsened glycaemic control and the escalation of therapy to exogenous insulin which further worsens bone density and promotes weight gain.

## Patient consent

Written consents have been obtained from the three patients whose hands and images are used in figures 1, 2 and 3.

## CRediT authorship contribution statement

**Harry Ward:** Writing – original draft, Investigation, Conceptualization. **Ali S. Jawad:** Writing – review & editing, Supervision, Methodology, Investigation, Conceptualization.

## Declaration of competing interest

The authors declare that they have no known competing financial interests or personal relationships that could have appeared to influence the work reported in this paper.
